# High-resolution crystal structure of the double nitrate hydrate [La(NO_3_)_6_]_2_[Ni(H_2_O)_6_]_3_·6H_2_O

**DOI:** 10.1107/S205698902400327X

**Published:** 2024-05-10

**Authors:** David Wenhua Bi, Yong Liu, Arnaud Magrez

**Affiliations:** a Crystal Growth Facility, Institute of Physics, École Polytechnique Fédérale de Lausanne (EPFL), Switzerland; University of Neuchâtel, Switzerland

**Keywords:** crystal structure, double nitrate, X-ray diffraction, hydrogen bonding

## Abstract

This study introduces bis­[hexa­kis­(nitrato-κ^2^ *O*,*O*′)lanthanum(III)] tris­[hexa­aqua­nickel(II)] hexa­hydrate, [La(NO_3_)_6_]_2_[Ni(H_2_O)_6_]_3_·6H_2_O, with a structure refined in the hexa­gonal space group *R*  . The salt com­prises [La(NO_3_)_6_]^3−^ icosa­hedra and [Ni(H_2_O)_6_]^2+^ octa­hedra, thus forming an intricate network of inter­penetrating honeycomb lattices arranged in layers. This arrangement is stabilized through strong hydrogen bonds. Two successive layers are connected *via* the second [Ni(H_2_O)_6_]^2+^ octa­hedra, forming sheets which are stacked perpendicular to the *c* axis and held in the crystal by van der Waals forces. The synthesis of [La(NO_3_)_6_]_2_[Ni(H_2_O)_6_]_3_·6H_2_O involves dissolving lanthanum(III) and nickel(II) oxides in nitric acid, followed by slow evaporation, yielding green hexa­gonal plate-like crystals.

## Abstract 2

Very large high-quality crystals of a new member of the family of double nitrates, namely, [La(NO_3_)_6_]_2_[Ni(H_2_O)_6_]_3_·6H_2_O, were crystallized in large amounts. The structure was determined *via* single-crystal X-ray diffraction to high resolution. Extensive structural information, including hydrogen-bonding details, was obtained at the same time.

## 1. Chemical context

**Figure 1:**
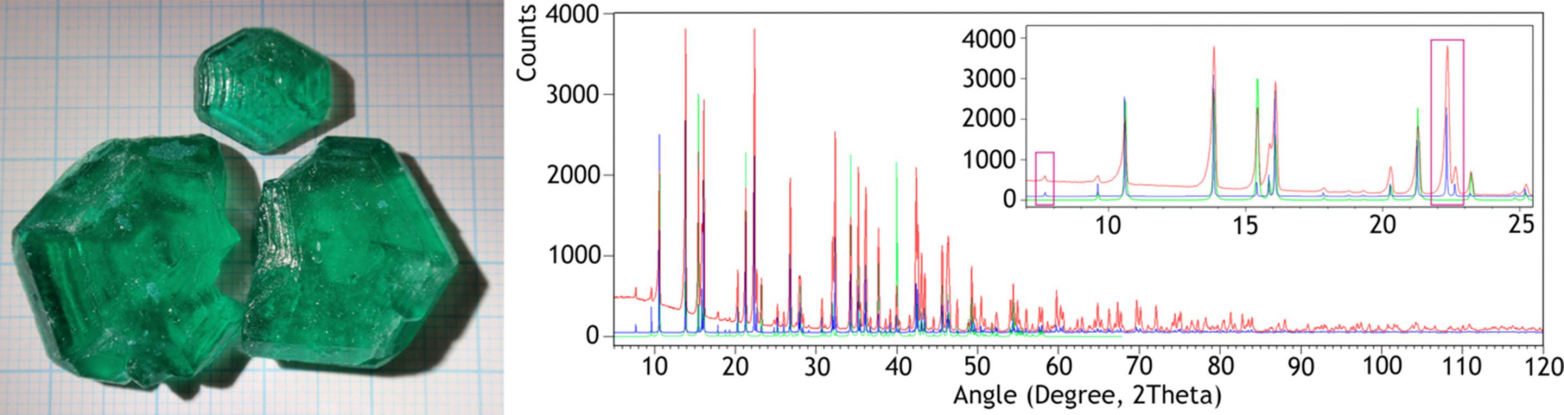
Left: large green crystals of [La(NO_3_)_6_]_2_[Ni(H_2_O)_6_]_3_·6H_2_O with a pseudo-hexa­gonal shape placed on a scale paper. The distance between two thick lines is 1 cm. Right: powder X-ray diffraction patterns of [La(NO_3_)_6_]_2_[Ni(H_2_O)_6_]_3_·6H_2_O. The PDF5 (ID = 00-049-1235) pattern available in the database is shown in green. The PXRD pattern calculated from the structure refined from single-crystal data and the experimental pattern measured on a Panalytical Empyrean diffractometer with Cu *K*α_1_ radiation (λ = 1.540596 Å) are shown in blue and red, respectively. In the inset, the high-intensity diffraction peaks absent in the PDF5 pattern are highlighted with purple rectangles.

Double nitrates, which contain two different metal cations and nitrate anions, have applications in various fields. They exhibit unique solubility properties, crystalline structures and special magnetic properties, and act as excellent precursors for the synthesis of mixed oxides. For example, double nitrates of Zn and Cu have been used to produce a mixed Cu and Zn oxide that exhibits high catalytic activity in reactions such as the water gas shift reaction to produce CO_2_ and H_2_ from CO and H_2_O (Smith *et al.*, 2010 ▸), as well as selective CO_2_ hydrogenation into methanol (Zhong *et al.*, 2020 ▸). Rare earth (RE) transition-metal (TM) double nitrates with the general formula RE_2_TM_3_(NO_3_)_12_·24H_2_O attracted much attention in the 1960s, in which the RE is a trivalent cation with an atomic number lower than that of Ho and the TM is a divalent cation, including Mg^2+^, Mn^2+^, Fe^2+^, Co^2+^, Ni^2+^, Cu^2+^ and Zn^2+^ (Hellwege & Hellwege, 1953 ▸; Brochard & Hellwege, 1953 ▸; Buckmaster *et al.*, 1968 ▸). It should be noted that when RE has a +4 oxidation state, double nitrates are isomorphic with the triclinic MgTh(NO_3_)_6_(H_2_O)_8_ salt (Šćavničar & Prodić, 1965 ▸). Cerium(III) magnesium and cerium(III) zinc double nitrates have been used extensively in nuclear orientation experiments because very low temperatures can be obtained by adiabatic demagnetization of the salt (Culvahouse, 1961 ▸). Their properties make them suitable magnetic thermometers (Thornley, 1963 ▸). Like ruby single crystals (Cr^3+^:Al_2_O_3_), a Ce-doped lanthanum magnesium double nitrate is an ideal medium to study phonon avalanche, a delayed and sudden relaxation of paramagnetic ions by the emission of phonons (Mims & Taylor, 1969 ▸). Heat capacity and susceptibility measurements sug­gested that Mn, Ni, Co and Cu lanthanum double nitrates show anti­ferromagnetic transitions below 0.5 K (Mess *et al.*, 1967 ▸, 1968 ▸). The lack of high-quality crystalline structures of these salts limits the profound understanding of the magnetic properties, as well as their theoretical investigation. We report herein on the growth of centimeter-large crystals of [La(NO_3_)_6_]_2_[Ni(H_2_O)_6_]_3_·6H_2_O (Fig. 1 ▸) and the crystal structure determined by single-crystal X-ray diffraction.

## 2. Structural commentary

**Figure 2:**
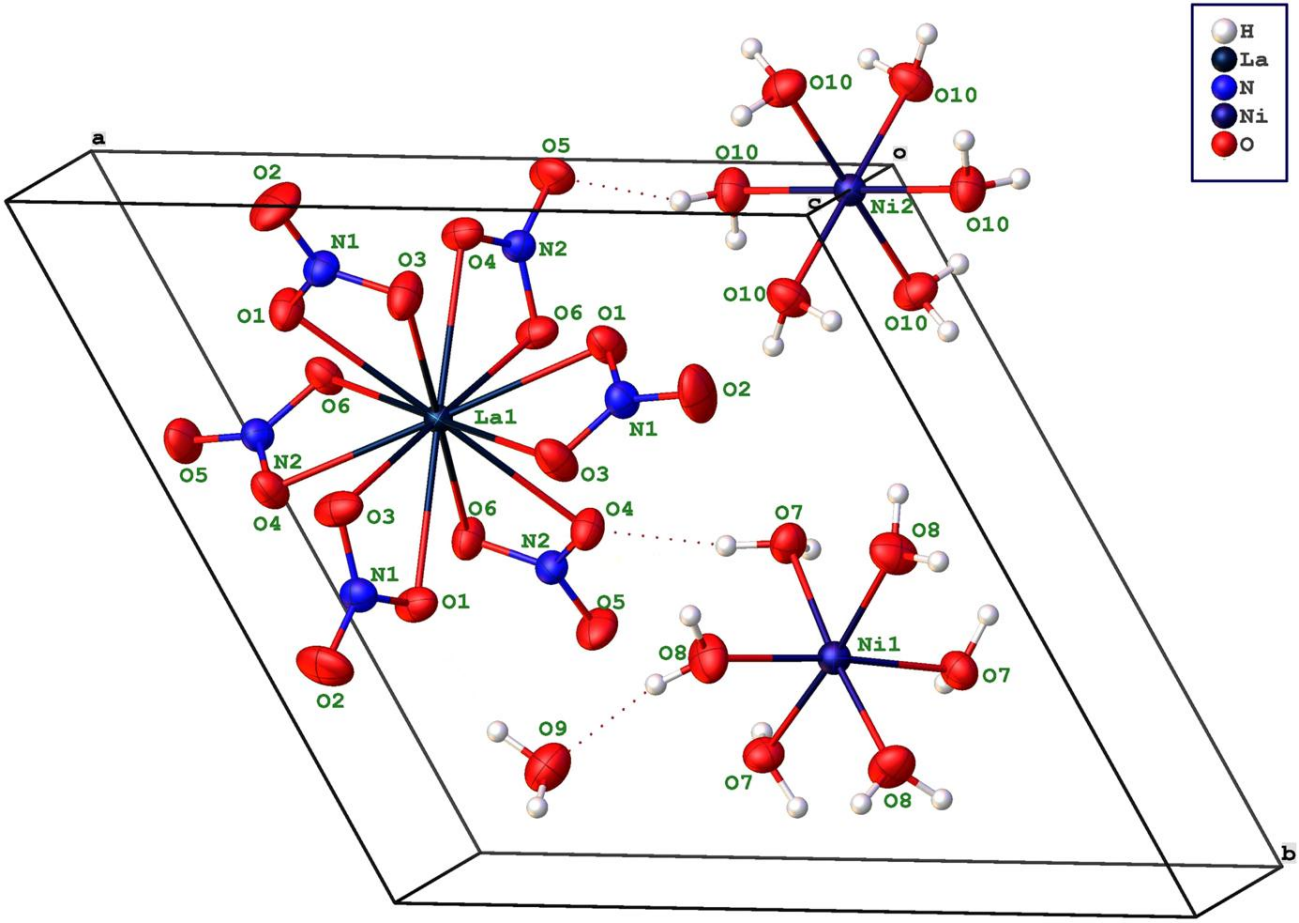
The mol­ecular structure of [La(NO_3_)_6_]_2_[Ni(H_2_O)_6_]_3_·6H_2_O, with displace­ment ellipsoids for all non-H atoms drawn at the 50% probability level. H atoms are represented by small spheres of arbitrary radius. H-atom labels have been omitted for clarity. The colour scheme for the different elements can be found in the legend. The viewing direction is slightly tilted from the *c* axis, in order to prevent overlap between atoms. Hydrogen bonds are indicated with thin dotted lines.

Similar to those found in the corresponding magnesium double salt, the title com­pound is made up of two types of ions, [Ni(H_2_O)_6_]^2+^ and [La(NO_3_)_6_]^3−^, which are linked together by hydrogen bonds with water mol­ecules in the structure. The La atom on the threefold axis is coordinated by 12 O atoms from six nitrate groups to form a slightly distorted icosa­hedron. The La—O distances range from 2.6339 (8) to 2.7012 (8) Å, which are com­parable to those found in La_2_Mg_3_(NO_3_)_12_·24H_2_O determined by neutron diffraction (Anderson *et al.*, 1977 ▸). As depicted in Fig. 2 ▸, the structure includes two crystallographically independent positions for Ni^2+^. Three water H7a—O7–H7b mol­ecules and three water H8a—O8—H8b mol­ecules surround Ni1, resulting in a distorted [Ni(H_2_O)_6_]^2+^ octa­hedron with *C*3 symmetry. In contrast, the Ni2-containing [Ni(H_2_O)_6_]^2+^ octa­hedron is highly symmetric, as Ni2 is situated in a site with 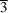 symmetry. The Ni—O bond lengths in both octa­hedra vary from 2.0471 (8) to 2.0531 (8) Å, similar to those found in [Ni(H_2_O)_6_](NO_3_)_2_ (Breternitz *et al.*, 2015 ▸).

**Figure 3:**
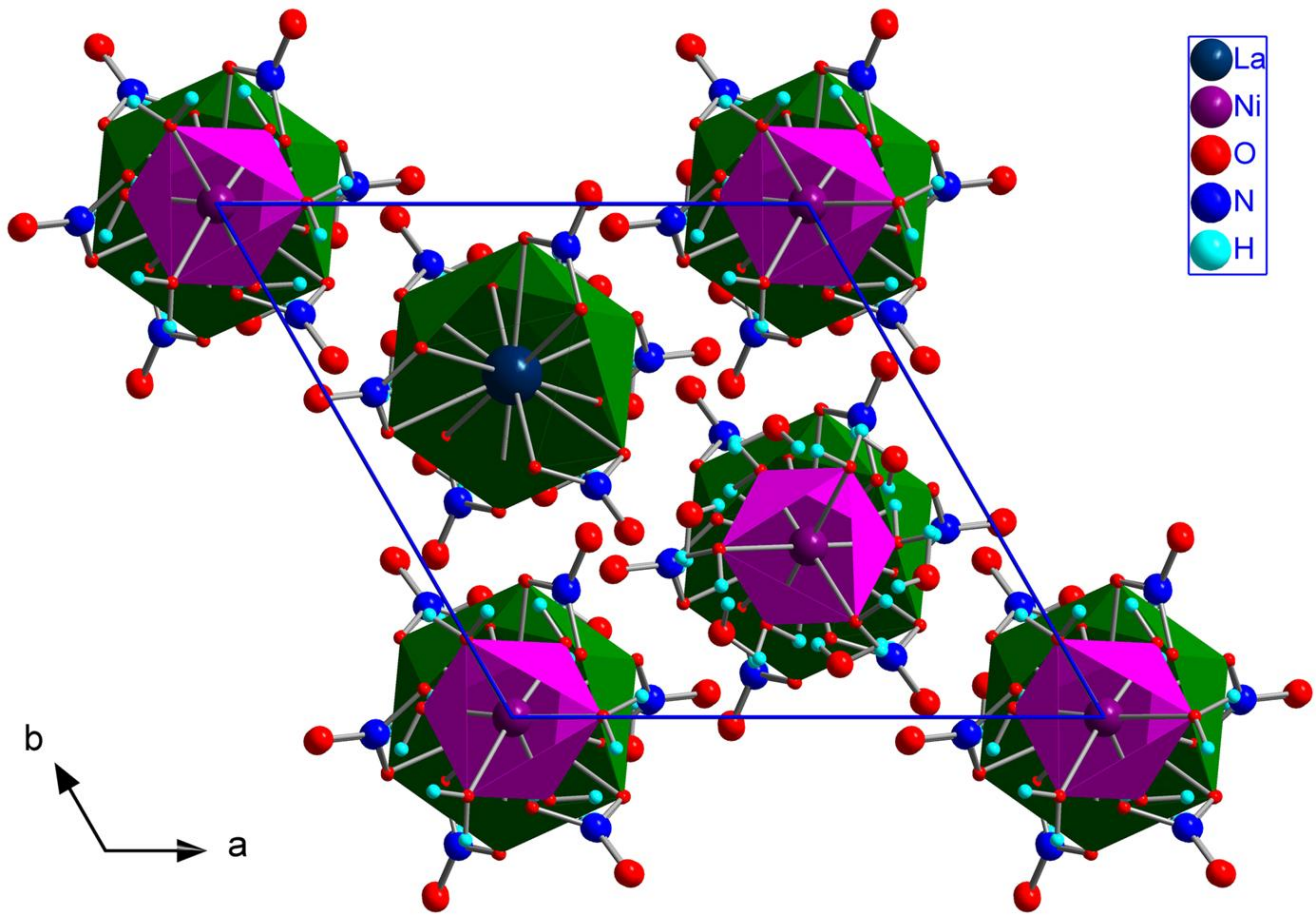
The [La(NO_3_)_6_]_2_[Ni(H_2_O)_6_]_3_·6H_2_O structure represented along the *c* axis. The colour scheme for the different elements of the structure can be found in the figure inset. [Ni(H_2_O)_6_]^2+^ octa­hedra and [La(NO_3_)_6_]^3−^ icosa­hedra are plotted as pink and green front-opening polyhedron, respectively. The unit-cell edges are plotted with blue lines.

**Figure 4:**
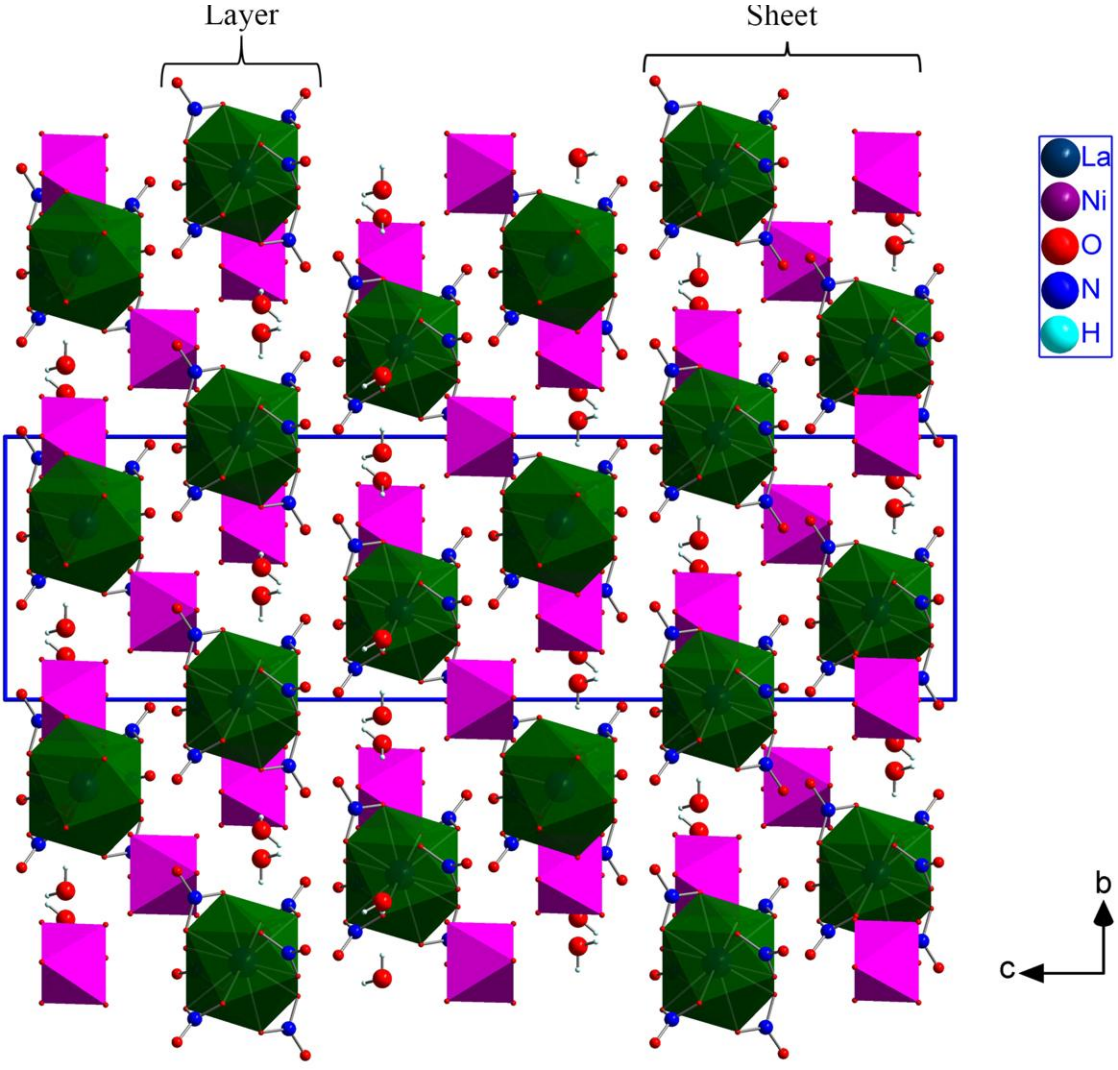
The [La(NO_3_)_6_]_2_[Ni(H_2_O)_6_]_3_·6H_2_O structure represented along the *a* axis. The colour scheme for the different elements of the structure can be found in the inset. [Ni(H_2_O)_6_]^2+^ octa­hedra and [La(NO_3_)_6_]^3−^ icosa­hedra are plotted as pink and green polyhedra, respectively. The unit-cell edges are plotted with blue lines.

As illustrated in Fig. 3 ▸, each [La(NO_3_)_6_]^3−^ icosa­hedron is surrounded by three Ni1-containing [Ni(H_2_O)_6_]^2+^ clusters, and each Ni1-containing octa­hedron is surrounded by three icosa­hedra. These two inter­penetrating honeycomb networks are arranged in a layer parallel to the *ab* plane. In this layer, the icosa­hedra are linked to the [Ni(H_2_O)_6_]^2+^ clusters through strong O7—H7*A*⋯O4, O9—H9*A*⋯O1 and O10—H10*A*⋯O9 hydrogen bonds. The six water H9*A*—O9—H9*B* mol­ecules per unit cell do not participate in the coordination of either La or Ni. Two successive layers are separated by Ni2-containing [Ni(H_2_O)_6_]^2+^ clusters, which bridge the layers between them *via* O10—H10*A*⋯O9 hydrogen bonds. The com­plex hydrogen-bonding network between the clusters is shown in Fig. 2 ▸ and the actual data for the hydrogen bonds are given in Table 1 ▸. The network of bonded clusters form sheets that are stacked perpendicular to the *c* axis (Fig. 4 ▸). The sheets are held together by van der Waals forces in the [La(NO_3_)_6_]_2_[Ni(H_2_O)_6_]_3_·6H_2_O structure.

## 3. Database survey

No record of the same com­pound was found in the Crystallography Open Database (COD) or the Inorganic Crystal Structure Database (ICSD). It is listed only once in the Powder Diffraction File (PDF) 2024 version, entry 00-049-1235, without any atomic positions provided. The powder X-ray diffraction (PXRD) pattern available in this database deviates significantly from both the theoretical pattern simulated from the structure refined *via* single-crystal XRD data and the experimental pattern recorded with powder obtained by crushing a few [La(NO_3_)_6_]_2_[Ni(H_2_O)_6_]_3_·6H_2_O single crystals. Notably, peaks below 10°, as well as those in the 22–23° region, are missing in the pattern found in PDF-00-49-1235 (Fig. 1 ▸).

## 4. Synthesis and crystallization

Lanthanum(III) oxide was dissolved in dilute HNO_3_ with a concentration of 1 mol l^−1^ (1 *M*) and nickel(II) oxide in dilute HNO_3_ with a concentration of 0.5 mol l^−1^ (0.5 *M*). In order to dissolve the nickel(II) oxide in the dilute HNO_3_, the solution was heated at 423 K over a period of 12 h until the nickel(II) oxide com­pletely dissolved and a green transparent solution was obtained. Lanthanum(III) oxide solution (0.2 l) was first mixed with nickel(II) oxide solution (1.2 l) and then 1 mol of citric acid was added to the mixture under vigorous stirring until com­plete dissolution. The solution was transferred to a fume hood for slow evaporation. After 30 d, green hexa­gonal plate-shaped crystals formed with different sizes, the maximum dimension being 2 cm.

## 5. Refinement

Crystal data, data collection and structure refinement details are summarized in Table 2 ▸. H atoms on O atoms were first located in a difference Fourier map and then refined isotropically in riding mode, with *U*_iso_(H) values of 1.5*U*_eq_ of the parent O atoms. The O—H distance was refined against the residual peaks, without further constraint.

## Supplementary Material

Crystal structure: contains datablock(s) I, global. DOI: 10.1107/S205698902400327X/tx2083sup1.cif

Structure factors: contains datablock(s) I. DOI: 10.1107/S205698902400327X/tx2083Isup2.hkl

CCDC reference: 2348374

Additional supporting information:  crystallographic information; 3D view; checkCIF report

## Figures and Tables

**Table 1:** Hydrogen-bond geometry (Å, °)

*D*—H⋯*A*	*D*—H	H⋯*A*	*D*⋯*A*	*D*—H⋯*A*
O8—H8*A*⋯O9	0.85	2.00	2.8386 (15)	169
O10—H10*A*⋯O9^i^	0.85	2.03	2.8149 (13)	153
O10—H10*B*⋯O5^ii^	0.86	2.14	2.9626 (13)	161
O7—H7*A*⋯O4^iii^	0.86	1.92	2.7656 (11)	168
O7—H7*B*⋯O6^iv^	0.86	2.13	2.9393 (11)	158

Symmetry codes: (i) 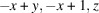 ; (ii) 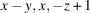 ; (iii) 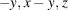 ; (iv) 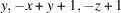 .

**Table 2:** Experimental details

Crystal data	
Chemical formula	[La(NO_3_)_6_]_2_[Ni(H_2_O)_6_]_3_·6H_2_O
*M*_r_	1630.45
Crystal system, space group	Hexagonal, *R* 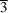
Temperature (K)	297
*a*, *c* (Å)	11.0230 (1), 34.4826 (4)
*V* (Å^3^)	3628.53 (8)
*Z*	3
Radiation type	Mo *K*α
μ (mm^−1^)	3.04
Crystal size (mm)	0.21 × 0.19 × 0.11

Data collection	
Diffractometer	XtaLAB Synergy-i HyPix3000
Absorption correction	Gaussian (*CrysAlis PRO*; Rigaku OD, 2023 ▸)
*T*_min_, *T*_max_	0.573, 1.000
No. of measured, independent and observed [*I* > 2σ(*I*)] reflections	27264, 2833, 2663
*R*_int_	0.023
(sin θ/λ)_max_ (Å^−1^)	0.746

Refinement	
*R*[*F* ^2^ > 2σ(*F* ^2^)], *wR*(*F* ^2^), *S*	0.013, 0.034, 1.06
No. of reflections	2833
No. of parameters	126
H-atom treatment	H-atom parameters constrained
Δρ_max_, Δρ_min_ (e Å^−3^)	0.30, −0.38

Computer programs: *CrysAlis PRO* (Rigaku OD, 2023 ▸), *SHELXT2018* (Sheldrick, 2015*a* ▸), *SHELXL2018* (Sheldrick, 2015*b* ▸), *DIAMOND* (Brandenburg, 2006 ▸) and *publCIF* (Westrip, 2010 ▸).

## supplementary crystallographic information

### Crystal data

**Table:**

[La(NO_3_)_6_]_2_[Ni(H_2_O)_6_]_3_·6H_2_O	*D*_x_ = 2.238 Mg m^−^^3^
*M**_r_* = 1630.45	Mo *K*α radiation, λ = 0.71073 Å
Hexagonal, *R*3	Cell parameters from 22661 reflections
*a* = 11.0230 (1) Å	θ = 2.2–36.2°
*c* = 34.4826 (4) Å	µ = 3.04 mm^−^^1^
*V* = 3628.53 (8) Å^3^	*T* = 297 K
*Z* = 3	Plate, blue
*F*(000) = 2430	0.21 × 0.19 × 0.11 mm

### Data collection

**Table:**

XtaLAB Synergy-i HyPix3000 diffractometer	2663 reflections with *I* > 2σ(*I*)
Radiation source: micro-focus sealed X-ray tube	*R*_int_ = 0.023
ω scans	θ_max_ = 32.0°, θ_min_ = 2.2°
Absorption correction: gaussian (CrysAlis PRO; Rigaku OD, 2023)	*h* = −16→16
*T*_min_ = 0.573, *T*_max_ = 1.000	*k* = −16→16
27264 measured reflections	*l* = −51→51
2833 independent reflections	

### Refinement

**Table:**

Refinement on *F*^2^	Hydrogen site location: difference Fourier map
Least-squares matrix: full	H-atom parameters constrained
*R*[*F*^2^ > 2σ(*F*^2^)] = 0.013	*w* = 1/[σ^2^(*F*_o_^2^) + (0.0161*P*)^2^ + 2.1621*P*] where *P* = (*F*_o_^2^ + 2*F*_c_^2^)/3
*wR*(*F*^2^) = 0.034	(Δ/σ)_max_ = 0.001
*S* = 1.06	Δρ_max_ = 0.30 e Å^−^^3^
2833 reflections	Δρ_min_ = −0.38 e Å^−^^3^
126 parameters	Extinction correction: SHELXL2018 (Sheldrick, 2015*b*), Fc^*^=kFc[1+0.001xFc^2^λ^3^/sin(2θ)]^-1/4^
0 restraints	Extinction coefficient: 0.00015 (2)

### Special details

**Table:**

Geometry. All esds (except the esd in the dihedral angle between two l.s. planes) are estimated using the full covariance matrix. The cell esds are taken into account individually in the estimation of esds in distances, angles and torsion angles; correlations between esds in cell parameters are only used when they are defined by crystal symmetry. An approximate (isotropic) treatment of cell esds is used for estimating esds involving l.s. planes.

### Fractional atomic coordinates and isotropic or equivalent isotropic displacement parameters (Å^2^)

**Table:**

	*x*	*y*	*z*	*U*_iso_*/*U*_eq_	
La1	0.666667	0.333333	0.58402 (2)	0.01660 (3)	
Ni1	0.333333	0.666667	0.59443 (2)	0.02146 (5)	
O1	0.82133 (9)	0.59977 (8)	0.60265 (2)	0.03067 (17)	
N1	0.88316 (10)	0.58535 (9)	0.63163 (3)	0.02614 (17)	
Ni2	0.000000	0.000000	0.500000	0.02201 (6)	
O2	0.97264 (11)	0.68693 (10)	0.64928 (3)	0.0487 (2)	
N2	0.45188 (9)	0.08678 (9)	0.53286 (2)	0.02312 (16)	
O3	0.84704 (10)	0.46142 (9)	0.64135 (2)	0.03567 (19)	
O10	0.14730 (9)	0.15389 (9)	0.53499 (2)	0.03423 (18)	
H10A	0.107682	0.185298	0.549969	0.051*	
H10B	0.202599	0.224634	0.521158	0.051*	
O9	0.76944 (11)	0.83079 (11)	0.60307 (3)	0.0422 (2)	
H9A	0.807994	0.887099	0.621866	0.063*	
H9B	0.807786	0.780452	0.602857	0.063*	
O4	0.51001 (9)	0.06874 (8)	0.56231 (2)	0.02866 (16)	
O5	0.36341 (10)	−0.01345 (9)	0.51426 (3)	0.03803 (19)	
O6	0.49052 (9)	0.21141 (8)	0.52368 (2)	0.02951 (16)	
O7	0.18944 (8)	0.68410 (8)	0.56094 (2)	0.02971 (16)	
H7A	0.114671	0.604003	0.559052	0.045*	
H7B	0.219721	0.704444	0.537602	0.045*	
O8	0.48738 (10)	0.66808 (11)	0.62819 (3)	0.0386 (2)	
H8A	0.572758	0.707366	0.621395	0.058*	
H8B	0.478129	0.605850	0.644424	0.058*	

### Atomic displacement parameters (Å^2^)

**Table:**

	*U*^11^	*U*^22^	*U*^33^	*U*^12^	*U*^13^	*U*^23^
La1	0.01709 (4)	0.01709 (4)	0.01561 (5)	0.00855 (2)	0.000	0.000
Ni1	0.02300 (7)	0.02300 (7)	0.01838 (10)	0.01150 (3)	0.000	0.000
O1	0.0387 (4)	0.0262 (4)	0.0281 (4)	0.0169 (3)	−0.0104 (3)	−0.0022 (3)
N1	0.0273 (4)	0.0246 (4)	0.0244 (4)	0.0113 (3)	−0.0047 (3)	−0.0029 (3)
Ni2	0.02193 (9)	0.02193 (9)	0.02215 (14)	0.01097 (4)	0.000	0.000
O2	0.0486 (6)	0.0326 (5)	0.0447 (5)	0.0052 (4)	−0.0219 (4)	−0.0080 (4)
N2	0.0238 (4)	0.0218 (4)	0.0237 (4)	0.0114 (3)	−0.0024 (3)	−0.0033 (3)
O3	0.0502 (5)	0.0268 (4)	0.0307 (4)	0.0198 (4)	−0.0134 (4)	−0.0016 (3)
O10	0.0303 (4)	0.0312 (4)	0.0322 (4)	0.0086 (3)	−0.0002 (3)	−0.0060 (3)
O9	0.0550 (6)	0.0493 (6)	0.0348 (5)	0.0353 (5)	0.0018 (4)	0.0013 (4)
O4	0.0368 (4)	0.0233 (3)	0.0258 (4)	0.0149 (3)	−0.0086 (3)	−0.0018 (3)
O5	0.0370 (4)	0.0285 (4)	0.0420 (5)	0.0115 (4)	−0.0174 (4)	−0.0113 (3)
O6	0.0382 (4)	0.0229 (3)	0.0281 (4)	0.0158 (3)	−0.0059 (3)	0.0002 (3)
O7	0.0262 (4)	0.0313 (4)	0.0279 (4)	0.0116 (3)	−0.0052 (3)	−0.0006 (3)
O8	0.0364 (4)	0.0502 (5)	0.0326 (4)	0.0244 (4)	−0.0028 (3)	0.0101 (4)

### Geometric parameters (Å, º)

**Table:**

La1—O1	2.6339 (8)	N1—O2	1.2217 (12)
La1—O1^i^	2.6340 (8)	N1—O3	1.2621 (12)
La1—O1^ii^	2.6340 (8)	Ni2—O10	2.0531 (8)
La1—O4	2.6481 (8)	Ni2—O10^v^	2.0531 (8)
La1—O4^ii^	2.6481 (8)	Ni2—O10^vi^	2.0531 (8)
La1—O4^i^	2.6481 (8)	Ni2—O10^vii^	2.0531 (8)
La1—O3	2.6546 (8)	Ni2—O10^viii^	2.0531 (8)
La1—O3^i^	2.6547 (8)	Ni2—O10^ix^	2.0531 (8)
La1—O3^ii^	2.6547 (8)	N2—O5	1.2271 (11)
La1—O6^ii^	2.7011 (8)	N2—O6	1.2585 (11)
La1—O6^i^	2.7011 (8)	N2—O4	1.2684 (11)
La1—O6	2.7012 (8)	O10—H10A	0.8536
Ni1—O7^iii^	2.0471 (8)	O10—H10B	0.8554
Ni1—O7^iv^	2.0471 (8)	O9—H9A	0.8499
Ni1—O7	2.0472 (8)	O9—H9B	0.8492
Ni1—O8	2.0526	O7—H7A	0.8576
Ni1—O8^iii^	2.0526	O7—H7B	0.8571
Ni1—O8^iv^	2.0526	O8—H8A	0.8489
O1—N1	1.2638 (11)	O8—H8B	0.8510
			
O1—La1—O1^i^	114.254 (14)	O4—La1—O6	47.44 (2)
O1—La1—O1^ii^	114.253 (14)	O4^ii^—La1—O6	111.22 (2)
O1^i^—La1—O1^ii^	114.252 (14)	O4^i^—La1—O6	70.19 (3)
O1—La1—O4	177.58 (2)	O3—La1—O6	177.63 (2)
O1^i^—La1—O4	67.33 (2)	O3^i^—La1—O6	111.60 (3)
O1^ii^—La1—O4	66.00 (2)	O3^ii^—La1—O6	110.71 (3)
O1—La1—O4^ii^	67.33 (2)	O6^ii^—La1—O6	67.04 (3)
O1^i^—La1—O4^ii^	66.00 (2)	O6^i^—La1—O6	67.04 (3)
O1^ii^—La1—O4^ii^	177.58 (2)	O7^iii^—Ni1—O7^iv^	91.31 (3)
O4—La1—O4^ii^	112.340 (15)	O7^iii^—Ni1—O7	91.31 (3)
O1—La1—O4^i^	66.00 (2)	O7^iv^—Ni1—O7	91.31 (3)
O1^i^—La1—O4^i^	177.58 (2)	O7^iii^—Ni1—O8	92.55 (4)
O1^ii^—La1—O4^i^	67.33 (2)	O7^iv^—Ni1—O8	85.37 (4)
O4—La1—O4^i^	112.341 (15)	O7—Ni1—O8	174.96 (4)
O4^ii^—La1—O4^i^	112.340 (15)	O7^iii^—Ni1—O8^iii^	174.96 (4)
O1—La1—O3	47.95 (2)	O7^iv^—Ni1—O8^iii^	92.55 (4)
O1^i^—La1—O3	72.20 (3)	O7—Ni1—O8^iii^	85.37 (3)
O1^ii^—La1—O3	115.50 (3)	O8—Ni1—O8^iii^	91.0
O4—La1—O3	134.33 (2)	O7^iii^—Ni1—O8^iv^	85.37 (4)
O4^ii^—La1—O3	66.91 (3)	O7^iv^—Ni1—O8^iv^	174.96 (4)
O4^i^—La1—O3	108.96 (3)	O7—Ni1—O8^iv^	92.55 (4)
O1—La1—O3^i^	115.51 (3)	O8—Ni1—O8^iv^	91.0
O1^i^—La1—O3^i^	47.95 (2)	O8^iii^—Ni1—O8^iv^	91.0
O1^ii^—La1—O3^i^	72.20 (3)	N1—O1—La1	98.15 (6)
O4—La1—O3^i^	66.91 (3)	O2—N1—O3	122.21 (10)
O4^ii^—La1—O3^i^	108.96 (3)	O2—N1—O1	121.18 (10)
O4^i^—La1—O3^i^	134.33 (2)	O3—N1—O1	116.61 (9)
O3—La1—O3^i^	70.62 (3)	O10—Ni2—O10^v^	180.0
O1—La1—O3^ii^	72.20 (3)	O10—Ni2—O10^vi^	91.03 (3)
O1^i^—La1—O3^ii^	115.50 (3)	O10^v^—Ni2—O10^vi^	88.97 (3)
O1^ii^—La1—O3^ii^	47.95 (2)	O10—Ni2—O10^vii^	88.97 (3)
O4—La1—O3^ii^	108.96 (3)	O10^v^—Ni2—O10^vii^	91.03 (3)
O4^ii^—La1—O3^ii^	134.33 (2)	O10^vi^—Ni2—O10^vii^	91.03 (3)
O4^i^—La1—O3^ii^	66.91 (3)	O10—Ni2—O10^viii^	91.03 (3)
O3—La1—O3^ii^	70.62 (3)	O10^v^—Ni2—O10^viii^	88.97 (3)
O3^i^—La1—O3^ii^	70.62 (3)	O10^vi^—Ni2—O10^viii^	88.97 (3)
O1—La1—O6^ii^	108.56 (3)	O10^vii^—Ni2—O10^viii^	180.00 (4)
O1^i^—La1—O6^ii^	66.37 (3)	O10—Ni2—O10^ix^	88.97 (3)
O1^ii^—La1—O6^ii^	130.25 (2)	O10^v^—Ni2—O10^ix^	91.03 (3)
O4—La1—O6^ii^	70.19 (3)	O10^vi^—Ni2—O10^ix^	180.00 (4)
O4^ii^—La1—O6^ii^	47.43 (2)	O10^vii^—Ni2—O10^ix^	88.97 (3)
O4^i^—La1—O6^ii^	111.22 (2)	O10^viii^—Ni2—O10^ix^	91.03 (3)
O3—La1—O6^ii^	111.60 (3)	O5—N2—O6	122.29 (9)
O3^i^—La1—O6^ii^	110.71 (3)	O5—N2—O4	120.90 (9)
O3^ii^—La1—O6^ii^	177.63 (2)	O6—N2—O4	116.80 (8)
O1—La1—O6^i^	66.37 (3)	N1—O3—La1	97.19 (6)
O1^i^—La1—O6^i^	130.25 (2)	Ni2—O10—H10A	109.7
O1^ii^—La1—O6^i^	108.56 (3)	Ni2—O10—H10B	109.5
O4—La1—O6^i^	111.22 (2)	H10A—O10—H10B	104.1
O4^ii^—La1—O6^i^	70.19 (3)	H9A—O9—H9B	104.7
O4^i^—La1—O6^i^	47.43 (2)	N2—O4—La1	98.97 (6)
O3—La1—O6^i^	110.71 (3)	N2—O6—La1	96.66 (6)
O3^i^—La1—O6^i^	177.63 (2)	Ni1—O7—H7A	109.7
O3^ii^—La1—O6^i^	111.60 (3)	Ni1—O7—H7B	109.6
O6^ii^—La1—O6^i^	67.04 (3)	H7A—O7—H7B	104.2
O1—La1—O6	130.25 (2)	Ni1—O8—H8A	123.3
O1^i^—La1—O6	108.57 (3)	Ni1—O8—H8B	127.0
O1^ii^—La1—O6	66.37 (3)	H8A—O8—H8B	104.4
			
La1—O1—N1—O2	177.19 (10)	O5—N2—O4—La1	−177.06 (9)
La1—O1—N1—O3	−3.22 (10)	O6—N2—O4—La1	3.67 (9)
O2—N1—O3—La1	−177.23 (10)	O5—N2—O6—La1	177.16 (9)
O1—N1—O3—La1	3.19 (10)	O4—N2—O6—La1	−3.58 (9)

Symmetry codes: (i) −*x*+*y*+1, −*x*+1, *z*; (ii) −*y*+1, *x*−*y*, *z*; (iii) −*y*+1, *x*−*y*+1, *z*; (iv) −*x*+*y*, −*x*+1, *z*; (v) −*x*, −*y*, −*z*+1; (vi) *x*−*y*, *x*, −*z*+1; (vii) −*y*, *x*−*y*, *z*; (viii) *y*, −*x*+*y*, −*z*+1; (ix) −*x*+*y*, −*x*, *z*.

### Hydrogen-bond geometry (Å, º)

**Table:**

*D*—H···*A*	*D*—H	H···*A*	*D*···*A*	*D*—H···*A*
O8—H8*A*···O9	0.85	2.00	2.8386 (15)	169
O10—H10*A*···O9^iv^	0.85	2.03	2.8149 (13)	153
O10—H10*B*···O5^vi^	0.86	2.14	2.9626 (13)	161
O7—H7*A*···O4^vii^	0.86	1.92	2.7656 (11)	168
O7—H7*B*···O6^x^	0.86	2.13	2.9393 (11)	158

Symmetry codes: (iv) −*x*+*y*, −*x*+1, *z*; (vi) *x*−*y*, *x*, −*z*+1; (vii) −*y*, *x*−*y*, *z*; (x) *y*, −*x*+*y*+1, −*z*+1.
